# Effect of tissue inhibitor of metalloproteinases-3 genetics polymorphism on clinicopathological characteristics of uterine cervical cancer patients in Taiwan

**DOI:** 10.7150/ijms.72378

**Published:** 2022-05-29

**Authors:** Jui-Fu Chung, Chia-Lin Chen, Yasser Nassef, Bei-Hao Shiu, Chun-Hao Wang, Fu-Hsuan Kuo, Yi-Hsuan Hsiao, Shun-Fa Yang, Po-Hui Wang

**Affiliations:** 1 Institute of Medicine, Chung Shan Medical University, Taichung, Taiwan; 2 Department of Radiology, Taichung Veterans General Hospital, Taichung, Taiwan; 3 Department of Obstetrics and Gynecology, Chung Shan Medical University Hospital, Taichung, Taiwan; 4 School of Medicine, Chung Shan Medical University, Taichung, Taiwan; 5 Department of Surgery, Chung Shan Medical University Hospital, Taichung, Taiwan; 6 Department of Medicine, National Taiwan University, Taipei, Taiwan; 7 Neurological Institute, Taichung Veterans General Hospital, Taichung, Taiwan; 8 Department of Obstetrics and Gynecology, Changhua Christian Hospital, Changhua, Taiwan; 9 Department of Medical Research, Chung Shan Medical University Hospital, Taichung, Taiwan

**Keywords:** tissue inhibitor of metalloproteinases-3, single nucleotide polymorphisms, uterine cervical cancer, lymph node metastasis, 5 years survival

## Abstract

Single nucleotide polymorphisms (SNPs) of tissue inhibitor of metalloproteinases-3 (*TIMP-3*) have been revealed to be related to various cancers. To date, no study explores the relationships between *TIMP-3* polymorphisms and uterine cervical cancer*.* The purposes of this research were to investigate the associations among genetic variants of *TIMP-3* and development and clinicopathological factors of uterine cervical cancer, and patient 5 years survival in Taiwanese women. The study included 123 patients with invasive cancer and 97 with precancerous lesions of uterine cervix, and 300 control women. *TIMP-3* polymorphisms rs9619311, rs9862 and rs11547635 were checked and their genotypic distributions were determined by real-time polymerase chain reaction. It showed that women with genotypes CT/TT in rs9862 were found to display a higher risk of developing cervical cancer with moderate and poor cell differentiation. Moreover, it revealed that cervical cancer patients carrying genotypes CC in rs9619311 exhibited a poorer 5 years survival, as compared to those with TT/TC in Taiwanese women, using univariate analysis. In addition, pelvic lymph node metastasis was determined to independently predict 5 years survival in cervical cancer patients using multivariate analysis. Conclusively, *TIMP-3* SNPs polymorphisms rs9619311 are related to cervical patient survival in Taiwanese women.

## Introduction

Matrix metalloproteinases (MMPs), capable of degrading extracellular matrix (ECM), are categorized to a zinc- as well as calcium-dependent endopeptidase family, and comprise 23 members 1. Based on their substrate specificity, the MMPs are classified into membrane-type matrix metalloproteinases (MT-MMPs), collagenases, gelatinases, stromelysins and matrilysins 2, 3. MMPs are involved in cell growth, invasion, survival, and adhesion in biological and pathological conditions, and then their activation can cause cell proliferation, invasion and metastasis of cancer cells 4-6. Tissue inhibitors of MMPs (TIMPs) are endogenous inhibitors of MMPs, and imbalance between the activities of MMPs and TIMPs may have an impact on cancer progression 7-10. The TIMPs are known as a family of at least four 20 to 29 kDa proteins (TIMPs 1-4) which reversibly inhibit the MMPs 11. TIMP-3 can inhibit the action of pro-MMP-2 that pertains to gelatinases, and is demonstrated to control cell death and suppress cancer cell invasion and metastasis 12-15. TIMP-3 overexpression was found to induce apoptosis in cancer cell lines of uterine cervix and breast 16.

Genetic polymorphisms are defined as allele base change in the shared DNA sequence of a gene where alternate alleles exceed 5% of some populations 17-19. When the genetic polymorphisms display a variation on the promoter area, exon or 3'-untranslated region of a gene, they may influence the gene expression, and then facilitate the development of disease and cancer 20, 21. Therefore, the genetic variations of *TIMP-3* in promoter area such as rs9619311 (-1296 T/C) and in the exon regions such as rs9862 (exon 3; 249 C/T) and rs11547635 (exon 3; 261 C/T) can be inferred to be associated with the occurrence and progression of uterine cervical cancer 22.

Uterine cervical cancer is the fourth most common cancer in female individuals in the world 23. It also ranked the eighth most common malignancy among women in 2014 according to the data of the Health Promotion Administration of the Ministry of Health and Welfare and Annual Cancer Registry Report in Taiwan. The age-standardized mortality rate of cervical cancer was calculated to 3.39 per 100000 women in this year, responsible for the seventh main cause of cancer mortality of females in the country. A continuous and multistep dysplasia progresses in cervical tumorigenesis 24, 25. Cervical intraepithelial neoplasia 1 (CIN 1) is referred to low-grade squamous intraepithelial lesion in cytological term (LSIL; also regarded as low-grade CIN or mild dysplasia in histologic counterpart), in which mitotic and immature cells only present in the lower third of the epithelium; whereas CIN 2 and CIN 3 are summed as high-grade CIN in histologic term and defined as high-grade squamous intraepithelial lesion in cytological counterpart, (HSIL) where mitotic and immature cells separately occur in the middle (CIN 2 or moderate dysplasia in histologic counterpart) as well as upper thirds of the epithelium or whole epithelium [severe dysplasia or carcinoma in situ (CIS), respectively, and summed as CIN 3 in histologic term], based on the Bethesda system. Near 90 % of CIN 1 regresses to normal, whereas, high-grade CIN progresses into invasive cancer in considerable rate, and is considered as cervical precancerous lesions 26, 27. Until now, no study investigates the involvement of *TIMP-3* genetic polymorphisms in the development of cervical cancer. Therefore, we conduct the study to associate the single nucleotide polymorphisms (SNPs) of *TIMP-3* with the carcinogenesis and progression of uterine cervical cancer, and further relate them to patient 5 years survival.

## Materials and Methods

### Female individual's enrollment

The research was conducted to consecutively enroll 123 women with cervical invasive cancer and 97 with precancerous lesions from the Department of Obstetrics and Gynecology in Chung Shan Medical University Hospital in Taichung, Taiwan, from February 1994 to February 2015. Meanwhile, 300 female subjects who received routine healthcare in the outpatient department of this hospital were recruited as the controls. They had no previous cervical cancer and precancerous lesions. The 123 cervical patients were staged according to the 2009 FIGO. The studied individuals were examined for the distribution of three *TIMP-3* genetic polymorphisms rs9619311, rs9862 and rs11547635*.* For the patients who had cervical cancer or precancerous lesions, cervical punch biopsy was done under colposcopy guidance and pathological findings were reported with final diagnosis of invasive cervical cancer, moderate, severe dysplasia or CIS. These patients were referred to cervical neoplasia group. The control group was defined as including female subjects without cervical neoplasias based on the normal cytological report from cervical Papanicolaou smear and further determined by normal colposcopic report in the general examination. All subjects were Taiwanese female individuals who resided in central Taiwan. The marital status and education level of cervical neoplasia group were well compatible with those of control group. Recruited patients underwent the standard treatment protocols, which conformed to guidelines offered by National Comprehensive Cancer Network. The Institutional Review Board in the Chung Shan Medical University Hospital agreed with this research (CSMUH number: CS18208). Informed consents were completed by all participants.

### Extraction of deoxyribonucleic acid (DNA) from the blood samples of all female individuals and determination of three TIMP-3 genetic polymorphisms

Laboratory technician performed venipuncture to obtain blood samples from all subjects. The samples were collected into Vacutainer tubes mixed with ethylenediaminetetraacetic acid, and then stored at 4°C. DNA was subsequently extracted from white blood cells in accordance with previous study 28, 29. The extracted DNA was dissolved in pH 7.8 TE buffer. Thereafter, it was quantified by the determination of OD260. The OD260/OD280 ratio was defined and the range of 1.8-2.0 was in line with our criteria and was determined as pure for preventing its cross reactivity from the current homologous RNA in the samples. The final products were refrigerated at -20°C and used as templates for the polymerase chain reaction (PCR).

According to the data of the international HapMap project and the previous research of Su et al., three genetic polymorphisms of *TIMP-3* rs9619311 (-1296 T/C), rs9862 (exon 3; 249 C/T), and rs11547635 (exon 3; 261 C/T) were determined 22. Genotyping of these 3 *TIMP-3* genetic variants was defined using the TaqMan SNP Genotyping Assay with an ABI StepOnePlus^TM^ Real-Time PCR System (Applied Biosystems, Foster City, CA, USA). The results were further checked with SDS version 3.0 software (Applied Biosystems, Foster City, CA, USA).

### Statistical analysis

Welch test was done and post hoc analysis was further performed by Dunnett test to compare the age distribution among patients with cervical invasive cancer and precancerous lesions, and normal controls. Chi-square or Fisher's tests were done to associate genotypic distributions of *TIMP-3* genetic polymorphisms with the incidence of cervical neoplasias. Age must be adjusted because the age of patients having precancerous lesions was inherently earlier than that of those with invasive cancer of uterine cervix. The *p* values were determined by chi-square or Fisher's exact tests and by logistic or multinomial logistic regression models for adjusting age. The adjusted odds ratios (AORs) with their 95% confidence intervals (CIs) were used to check the relationships among genotypic distributions of TIMP-3 genetic variants and the incidence of cervical neoplasias (including precancerous lesions and invasive cancer) by the logistic or multinomial logistic regression models for adjusting age. Chi-square or Fisher's exact tests were applied to associate *TIMP-3* SNPs with clinicopathological characteristics including clinical stage, pathologic type, cell grading, stromal invasion depth and tumor diameter of cancer masses, as well as invasions of parametrium and vagina, and pelvic lymph node metastasis. In univariate analysis, the Kaplan-Meier curve model was performed to determine the significances of *TMIP-3* genetic variants and clinicopathological parameters for patient survival relative to survival time, which were related to 5 years survival of cervical cancer patients. The log-rank test was applied to check the differences among them. In multivariate analysis, the influences of *TIMP-3* SNPs and these clinicopathological variables on 5 years survival of the patients were assessed using the Cox proportional hazard model in correspondence to survival time. The hazard ratios (HRs) were then determined. The SPSS, version 18.0 and WinPepi Software, version 10.0 were performed for statistical analysis. *P* <0.05 was defined to have statistically significant difference.

## Results

Patients with cervical neoplasm were significantly different from control women in the age distribution (50.8 ± 13.8 vs. 44.0 ± 10.0, *p*<0.001). Using the Welch test by Dunnett test as post hoc analysis, the age distributions were statistically different between patients with cervical cancer and those with precancerous lesions (56.2 ± 12.6 vs. 43.9 ± 12.3, *p*<0.001), and between patients with cervical cancer and control individuals (56.2 ± 12.6 vs. 44.0 ± 10.0, *p*<0.001). Whereas, no statistical difference was found in the age distribution between patients with precancerous lesions and control subjects (43.9 ± 12.3 vs. 44.0 ± 10.0, *p*= 1.000).

The genotypic frequencies of *TIMP-3* polymorphisms rs9619311 accorded with Hardy-Weinberg equilibrium in the normal controls [χ^2^ value: 0.69, *p*=0.709, degree of freedom (d.f.)=2]. The genotypic distributions of other *TIMP-3* genetic polymorphisms rs9862 and rs11547635 were all in line with the equilibrium (χ^2^ value: 3.84, *p*=0.147, d.f.=2 and χ^2^ value: 0.09,* p*=0.958, d.f.=2, respectively).

It showed no significant differences for the genotypic distributions of T/T, T/C and C/C in *TIMP-3* polymorphism rs9619311 (*p*=0.620) between patients with cervical neoplasias and control females. The genotypic frequencies of other *TIMP-3* SNPs, rs9862 and rs11547635 also revealed no significant differences between patients with cervical neoplasias and control women (*p*=0.655 and 0.774, respectively; Table 1). After adjusting for age, it still found no significant differences in these three *TIMP-3* genetic variants between these patients and control women (*p*=0.783, 0.618 and 0.789, respectively; Table 1).

While patients with cervical neoplasias group was further subdivided into subgroup of patients with invasive cancer and subgroup of those with precancerous lesions, it still did not reveal significant differences for the genetic frequencies of T/T, T/C and C/C in *TIMP-3* genetic polymorphism rs9619311 among invasive cancer, precancerous and control subgroups (*p*=0.171; Table 2). No significantly different distributions were also found in *TIMP-3* genetic polymorphisms rs9862 and rs11547635 (*p*=0.551 and *p*=0.969, respectively; Table 2). After adjusting for age, there were still no significant differences in* TIMP-3* rs9619311 among them (T/C vs T/T, *p*=0.137; C/C vs T/T, unavailable*,* while precancerous lesions subgroup compared with normal control subgroup; T/C vs T/T, *p*=0.227; C/C vs T/T, 0.838*,* while invasive cancer subgroup compared with normal control; Table 2). It also found no significant differences in the genotypic frequencies of other *TIMP-3* genetic polymorphisms, rs9862 (C/T vs C/C, *p*=0.909; T/T vs C/C, *p*=0.145*,* while precancerous lesions subgroup compared with normal control; C/T vs C/C, *p*=0.373; T/T vs C/C, 0.494*,* while invasive cancer subgroup compared with normal control; Table 2) and rs11547635 (C/T vs C/C, *p*=0.683; T/T vs C/C, *p*=0.974*,* while precancerous lesions subgroup compared with normal control; C/T vs C/C, *p*=0.619; T/T vs C/C, 0.919*,* while invasive cancer subgroup compared with normal control; Table 2). The risks of invasive cancer and precancerous lesion of uterine cervix were not elevated for these *TIMP-3* genetic polymorphisms (Table 2).

Compared with CC, patients with cervical cancer carrying genotypes CT/TT in *TIMP-3* rs9862 had a higher risk of developing moderate and poor cell grading (grade 2/3, OR: 3.61, 95% CI: 1.14-12.01,* p*=0.012; Table 3). There were no associations of *TIMP-3* rs9862 with other clinicopathological characteristics. Moreover, there were no relationships among *TIMP-3* genetic variants rs9619311, rs11547635 and various clinicopathological parameters.

**Figure 1:**
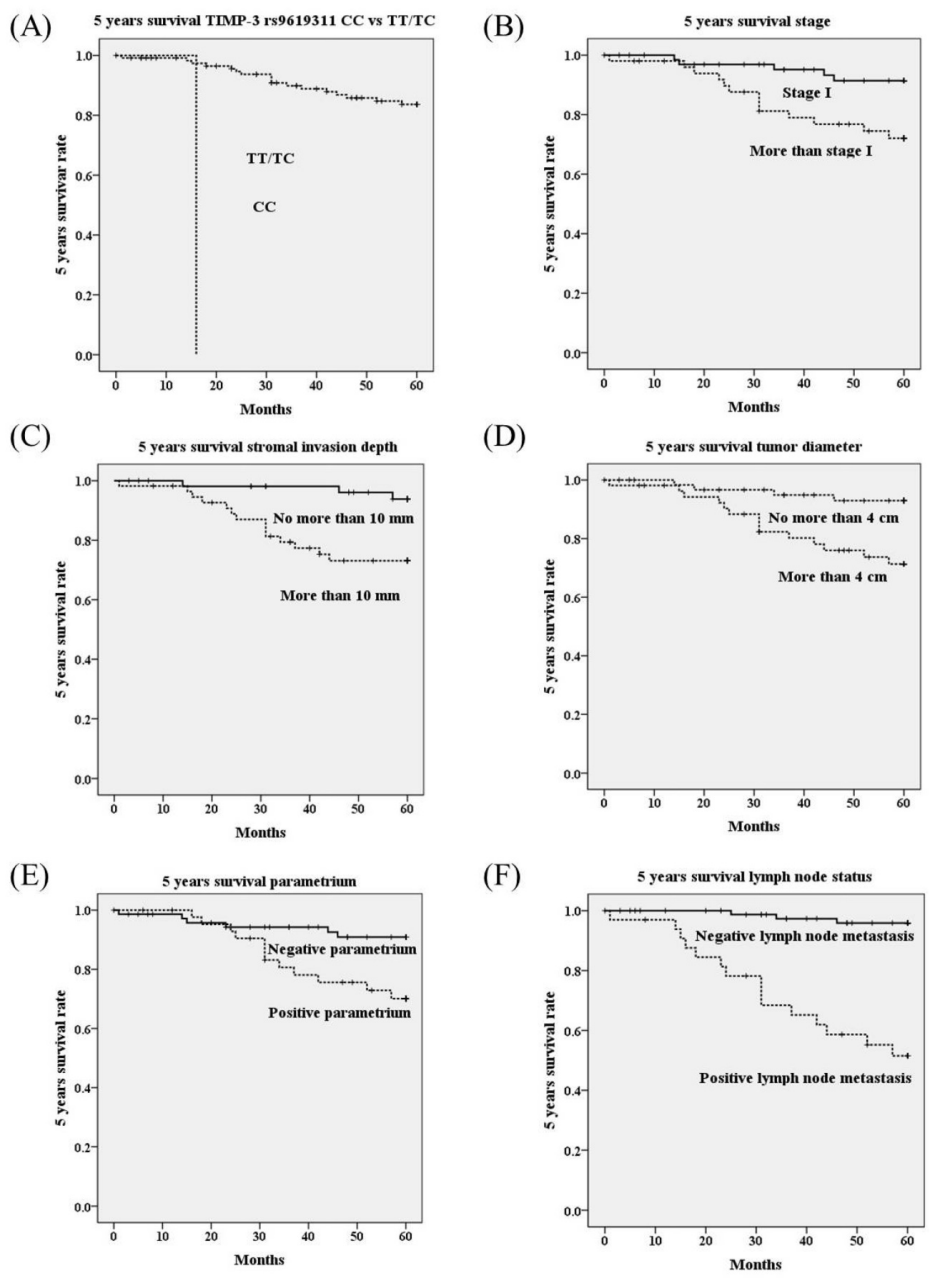
Five years survival rate based on polymorphisms of tissue inhibitor of metalloproteinases-3 (TIMP-3) (A), stage (B), stromal invasion depth (C), tumor diameter (D), parametrium invasion (E) and lymph node status (F) using Kaplan-Meier curve. Significantly worse 5 years survivals are showed in cervical cancer patients with genotype CC than those with TT/TC in TIMP-3 polymorphism rs9619311 (*p*<0.001 by log rank test), with stage ≥ stage II than stage I (*p*=0.009), with stromal invasion depth >10 mm than ≤10 mm (*p*=0.003), with tumor diameter >4 cm than ≤4 cm (*p*=0.004), with positive parametrium than negative parametrium invasion (*p*=0.009), with positive lymph than negative lymph node metastasis (*p*<0.001).

Using univariate analysis, genotype CC in *TIMP-3* genetic polymorphism rs9619311 had poorer 5 years survival as compared to genotypes TT/TC (OR=37.05, 95% CI=3.85-356.26;* p*<0.001; Table 4; Figure 1A). Other *TIMP-3* variants rs9862 and rs11547635 were not associated with 5 years survival. Furthermore, it revealed that HRs with poorer 5 years survival could be noted in cervical patients with more advanced stages (≥ stage II; *p*=0.009; Figure 1B), deep stromal invasion (*p*=0.003; Figure 1C), large tumor diameter (*p*=0.004; Figure 1D), positive parametrium invasion (*p*=0.009; Figure 1E), and positive pelvic lymph node metastasis (*p*<0.001; Table 4; Figure 1F). Using multivariate analysis, *TIMP-3* genetic polymorphisms rs9619311, rs9862 and rs11547635 were not related to 5 years survival. Whereas, only positive lymph node metastasis could significantly predict poorer 5 years survival among clinicopathological variables (HR: 6.84, 95% CI: 1.41-33.11, *p*=0.017; Table 5).

## Discussion

TMIP-3 has its unique binding to the extracellular matrix and is found to inhibit all MMPs, especially MMP-2 and MMP-9 12, 30, 31. Decreased TMIP-3 expression has been found in tissues of several types of cancer, as compared to their normal controls 14, 32, 33. It was also demonstrated to induce apoptosis in cervical cancer 16. Reduced TMIP-3 expression may be associated with poor outcome, including large tumor size, poor stage and positive metastasis 22, 34, 35. *TIMP-3* genetic variant rs9619311 is at promoter region (cDNA position and nucleotide change, -1296 T/C), rs9862 is at exon region (synonymous SNPs, 249C/T: H83H), and rs11547635 is at exon as well (synonymous SNPs, 261C/T; S87S) 22. These genetic polymorphisms may influence *TIMP-3* expressions and have been demonstrated to be associated with susceptibility and progression of several cancers 20, 21, 36-38. To date, no study investigates the implications of *TIMP-3* genetic polymorphisms in the occurrence of cervical cancer and patient prognosis in Taiwanese women. Therefore, we devised this study to explore the significance of *TIMP-3* genetic polymorphisms in the development and progression of cervical cancer.

This study was conducted to associate the genotypic distributions of three *TIMP-3* genetic polymorphisms, rs9619311, rs9862 and rs11547635, with uterine cervical carcinogenesis in Taiwanese women. It revealed that there were no significantly different genotypic frequencies in these three *TIMP-3* polymorphisms between patients with cervical neoplasias and normal control women. Even after classifying cervical neoplasia patients group into invasive and precancerous subgroups and controlling for age, there were still no statistically different genotypic distributions of these *TIMP-3* polymorphisms. *TIMP-3* genetic variants, rs9619311, rs9862 and rs11547635, were not found to be related to the susceptibility of cervical cancer. However among these three *TIMP-3* variants, Su et al could find that Taiwanese carrying allele T in rs9862 displayed more risk of developing oral squamous cell carcinoma 22. In addition, Tsai et al. revealed that *TIMP-3* rs961033 at promoter region were significantly related to the occurrence of hepatocellular carcinoma among women but not in men 38. In contrast, genotypic frequencies in *TIMP-3* rs9619311 were not associated with the development of bladder cancer 39.

In relating the *TIMP-3* genetic variants rs9619311, rs9862 and rs11547635 to the clinicopathological parameters, only women with CT/TT in rs9862 were found to display a higher risk of developing cervical cancer with moderate and poor cell differentiation. Otherwise, no TIMP-3 genotypes in these polymorphisms were demonstrated to be related to the clinicopathological factors. Reduced TIMP-3 expression has been reported to result in large tumor size, advanced stage, and metastasis 22, 34, 35. Interestingly, Chang et al. revealed that patients carrying T/T in *TIMP-3* rs9862 exhibited a higher risk of presenting mutant epidermal growth factor receptor (EGFR), as compared to those with CC, in lung adenocarcinoma. In addition, among male subjects having mutant EGFR, patients carrying allele T in *TIMP-3* rs9862 exhibited a higher risk of displaying an advanced stage and positive lymph node metastasis 40.

Because *TIMP-3* genetic variants have been reported to be correlated with breast cancer prognosis 41, 42, we further investigated the association of *TIMP-3* SNPs with patient survival in cervical cancer. The current study reveals that cervical cancer patients carrying genotypes CC in rs9619311 exhibit a poorer 5 years survival, as compared to those with TT/TC in Taiwanese women, using univariate analysis. But, the low minor allele C frequency, which led to small numbers of homozygote CC women, restricted this conclusion. Other *TIMP-3* SNPs were found to be not associated with patient 5 years survival of cervical cancer patients. Bashash et al. found that genetic variants in rs9619311 were not related to patient survival in patients with gastroesophageal junction cancer 36. In contrast, patients carrying CT/TT displayed a poorer survival in rs9862 even after adjusting for age, disease stage and treatment, as compared to those with CC. However, in multivariate analysis in this current research, it reveals that *TIMP-3* polymorphisms rs9619311, rs9862 and rs11547635 are not associated with patient survival in cervical cancer for Taiwanese women after adjusting the clinicopathological variables. Moreover, only pelvic lymph node metastasis was determined to independently predict 5 years survival in cervical cancer patients among these parameters in Taiwan. This finding is reasonable since pelvic lymph node metastasis has been demonstrated to be the most important indicator to predict survival of cervical cancer patients 43.

Although this study is the only research investigating the relationships between *TIMP-3* SNPs and cervical cancer, there are several limitations present. First, the study was a hospital-based study and the selection bias may inevitably occur. Second, only central Taiwan subjects were included and residents from other regions were not enrolled. In addition, the studied sample size may not be large enough to draw a statistical significance especially for low subject numbers in precancerous subgroup and low minor allele C frequency in rs9619311, which resulted in small numbers of homozygote CC women, and therefore restricted the external validity of this research. Third, female subjects in the control subgroup were recruited from the outpatient clinic of our hospital for general examination. Routine examination for human papillomavirus (HPV) infection was not popular for studied female individuals even after suggestion by medical staff because of their conservative attitude. The effect of HPV cannot be analyzed. Fourth, since the occurrence ages of cervical precancerous lesions and invasive cancer are inherently different, the age distributions of both diseases are different. Therefore, the logistic regression models were needed to adjust the impact of age. Fifth, we did not collect the tissue samples in the enrolled women, we therefore could not investigate the possible correlation between *TIMP-3* polymorphisms and TIMP-3 levels in the women. However, we shall investigate any possible correlation in the future study.

## Figures and Tables

**Table 1:** Genotypic distributions of three tissue inhibitor of metalloproteinases-3 polymorphisms in patients with cervical neoplasias and normal controls in Taiwanese women

Genetic variants	Normal controls (n = 300)	Cervical neoplasias^a^ (n= 220 )	ORs (95% CIs)	*p* values	AORs (95% CIs)^b^	Adjusted *p* values^b^
**rs9619311**				0.620		0.783
T/T^c^	246	181	1.00		1.00	
T/C	50	38	1.03 (0.65-1.64)	0.891	1.06 (0.65-1.71)	0.829
C/C	4	1	0.34 (0.04-3.07)	0.336	0.47 (0.05-4.42)	0.512
T/T^c^	246	181	1.00		1.00	
T/C & C/C	54	39	0.98 (0.62-1.55)	0.936	1.02 (0.63-1.63)	0.942
T/T & T/C^c^	296	219	1.00		1.00	
C/C	4	1	0.34 (0.04-3.04)	0.333	0.47 (0.05-4.37)	0.506
**rs9862**				0.655		0.618
C/C^c^	110	78	1.00		1.00	
C/T	130	98	1.06 (0.72-1.57)	0.759	1.10(0.73-1.66)	0.642
T/T	57	34	0.84 (0.50-1.41)	0.510	0.85 (0.50-1.4 6)	0.558
C/C^c^	110	78	1.00		1.00	
C/T & T/T	187	132	1.00 (0.69-1.44)	0.981	1.03 (0.70-1.50)	0.900
C/C & C/T^c^	240	176	1.00		1.00	
T/T	57	34	0.81(0.51-1.30)	0.386	0.81 (0.50-1.31)	0.388
**rs11547635**				0.774		0.789
C/C^c^	127	86	1.00		1.00	
C/T	127	98	1.14 (0.78-1.67)	0.500	1.14 (0.77-1.70)	0.517
T/T	34	23	1.00 (0.55-1.81)	0.997	1.00 (0.53-1.87)	0.997
C/C^c^	127	86	1.00		1.00	
C/T & T/T	161	121	1.11 (0.77-1.59)	0.572	1.11 (0.76-1.63)	0.586
C/C & C/T^c^	254	184	1.00		1.00	
T/T	34	23	0.93 (0.53-1.64)	0.811	0.93 (0.52-1.69)	0.820

Statistical analysis: logistic regression model or chi-square or Fisher's tests. ^a^Cervical neoplasias consisted of precancerous lesions and invasive cancer of uterine cervix. ^b^The adjusted *p* values and adjusted odds ratios (AORs) and their 95% confident intervals (95% CIs) were defined through logistic regression model after adjusting age. ^c^Used as a reference for comparison to define the odds ratios of other genotypic distributions.

**Table 2:** Genotypic distributions of three tissue inhibitor of metalloproteinases-3 polymorphisms in patients with uterine cervical invasive cancer or precancerous lesion and normal controls in Taiwanese women

Genetic variants	Normal controls (n = 300)	Pre-cancerous lesions (n = 97)	Invasive cancer (n = 123)	*p* values	AORs (95% CIs)^a^	Ad. *p* values	AORs (95% CIs)^b^	Ad. *p* values
**rs9619311**								
T/T^c^	246	74	107	0.171	1.00		1.00	
T/C	50	23	15		1.53 (0.87-2.67)	0.137	0.66 (0.33-1.30)	0.227
C/C	4	0	1		u.a.	u.a.	1.27 (0.13-12.94)	0.838
T/T^c^	246	74	107	0.120	1.00		1.00	
T/C & C/C	54	23	16		1.41 (0.81-.046)	0.219	0.68 (0.35-1.31)	0.247
T/T & T/C^c^	296	97	122	0.829	1.00		1.00	
C/C	4	0	1		u.a.	u.a.	1.35 (0.13-13.71)	0.798
**rs9862**								
C/C^c^	110	37	41	0.551	1.00		1.00	
C/T	130	45	53		1.03 (0.62-1.70)	0.909	1.28 (0.75-2.19)	0.373
T/T	57	11	23		0.57 (0.27-1.21)	0.145	1.27 (0.64-2.49)	0.494
C/C^c^	110	37	41	0.779	1.00		1.00	
C/T & T/T	187	56	76		0.89 (0.55-1.44)	0.636	1.27 (0.77-2.11)	0.346
C/C & C/T^c^	240	82	94	0.233	1.00		1.00	
T/T	57	11	23		0.57 (0.28-1.13)	0.106	1.10 (0.61-2.01)	0.748
**rs11547635**								
C/C^c^	127	38	48	0.969	1.00		1.00	
C/T	127	42	56		1.11 (0.67-1.84)	0.683	1.14 (0.68-1.91)	0.619
T/T	34	10	13		0.99 (0.45-2.18)	0.974	0.96 (0.43-2.15)	0.919
C/C^c^	127	38	48	0.840	1.00		1.00	
C/T & T/T	161	52	69		1.09 (0.67-1.75)	0.740	1.10 (0.68-1.80)	0.701
C/C & C/T^c^	254	80	104	0.972	1.00		1.00	
T/T	34	10	13		0.94 (0.44-1.98)	0.861	0.90 (0.42-1.92)	0.780

^a^Adjusted *p* values and adjusted odds ratios with their 95% CIs were evaluated using multinomial logistic regression models after adjusting for age between patients with uterine cervical precancerous lesions and control women. ^b^Adjusted *p* values and adjusted odds ratios with their 95% CIs were evaluated using multinomial logistic regression models after adjusting for age between patients with uterine cervical invasive cancer and control women. ^c^Used as a reference for comparison to assess the odds ratios of other genotypic distributions. AORs, adjusted odds ratios; 95% CIs, 95% confidence intervals; Ad. *p*, adjusted *p*; u.a., unavailable.

**Table 3:** Associations between genotypic distributions of tissue inhibitor of metalloproteinases-3 polymorphisms and clinicopathological factors in patients with cervical invasive cancer

Parameters^a^	rs9619311				rs9862				rs11547635			
	TT^b^	TC/CC	TT/TC^b^	CC	CC^b^	CT/TT	CC/CT^b^	TT	CC^b^	CT/TT	CC/CT^b^	TT
**Clinical stage**												
stage I^b^	61	9	70	0	22	47	53	16	29	39	62	6
≥ stage II	45	7	51	1	19	28	40	7	18	30	40	8
*P* value	0.922		0.426		0.345		0.271		0.578		0.202	
**Pathologic type**												
squamous cell carcinoma^b^	93	15	107	1	34	70	82	22	44	61	93	12
adenocarcinoma	14	1	15	0	7	6	12	1	3	9	10	2
* P* value	0.690		0.708		0.215		0.459		0.357		0.636	
**Cell grading**												
well (grade 1)^b^	14	5	19	0	11	7	16	2	5	12	13	4
moderate & poor (grades 2/3)	93	11	103	1	30	69	78	21	42	58	90	10
* P* value	0.073		1.000		0.012^*^		0.520		0.328		0.121	
**Stromal invasion depth**												
≤10 mm^b^	50	9	59	0	23	35	45	13	20	36	50	6
>10 mm	51	5	55	1	14	37	41	10	24	29	47	6
*P* value	0.300		0.487		0.179		0.720		0.309		0.919	
**Tumor diameter**												
≤ 4 cm^b^	56	10	66	0	24	41	53	12	25	39	57	7
>4 cm	49	6	54	1	17	33	39	11	22	29	44	7
* P* value	0.493		0.455		0.746		0.638		0.659		0.650	
**Parametrium**												
no invasion^b^	67	9	76	0	26	48	59	15	27	45	64	8
invasion	38	7	44	1	15	26	33	8	20	23	37	6
* P* value	0.560		0.372		0.876		0.922		0.342		0.652	
**Vagina**												
no invasion^b^	68	10	78	0	27	48	61	14	30	44	66	8
invasion	37	6	42	1	14	26	31	9	17	24	35	6
* P* value	0.860		0.355		0.915		0.625		0.923		0.563	
**Pelvic lymph node**												
no metastasis^b^	75	13	88	0	32	54	72	14	35	49	75	9
metastasis	30	3	32	1	9	20	20	9	12	19	26	5
* P* value	0.553		0.273		0.548		0.108		0.775		0.521	

Statistical analyses: chi-square or Fisher's exact tests ^*^p<0.05 ^a^Clinicopathological data of some cases could not be obtained from the patients with cervical invasive cancer because of incomplete medical charts or records. ^b^As a reference.

**Table 4:** Univariate analysis of genetic polymorphisms of tissue inhibitor of metalloproteinases-3 and clinicopathological variables for 5 years survival in cervical cancer patients

	5 years survival			
Variables^a^	+	-	*P* value	HR (95% CIs)^c^
**rs9619311**				
TT^b^	88	16	0.768	1.00
TC/CC	14	2		0.80 (0.18-3.49)
TT/TC^b^	102	17	<0.001^*^	1.00
CC	0	1		37.05 (3.85-356.26)
**rs9862**				
CC^b^	33	8	0.329	1.00
CT/TT	65	8		0.62 (0.23-1.65)
CC/CT^b^	77	13	0.995	1.00
TT	19	3		1.00 (0.28-3.50)
**rs11547635**				
CC^b^	41	3	0.066	1.00
CT/TT	56	14		3.03 (0.87-10.56)
CC/CT^b^	85	15	0.889	1.00
TT	12	2		0.90 (0.21-3.94)
**Clinical stage**				
stage I^b^	62	5	0.009^*^	1.00
≥ stage II	39	13		3.63 (1.30-10.19)
**Pathologic type**				
squamous cell carcinoma^b^	92	14	0.160	1.00
adenocarcinoma	10	4		2.17 (0.72-6.60)
**Cell grading**				
well (grade 1)^b^	15	3	0.941	1.00
moderate & poor (grades 2/3)	87	15		0.95 (0.28-3.30)
**Stromal invasion depth**				
≤ 10 mm^b^	53	3	0.003^*^	1.00
> 10 mm	42	14		5.29 (1.52-18.42)
**Tumour diameter**				
≤ 4 cm^b^	59	4	0.004^*^	1.00
> 4 cm	41	14		4.45 (1.47-13.54)
**Parametrium**				
no invasion^b^	67	6	0.009^*^	1.00
invasion	33	12		3.43 (1.29-9.13)
**Vagina**				
no invasion^b^	66	9	0.131	1.00
invasion	34	9		2.01 (0.80-5.07)
**Pelvic lymph node**				
no metastasis^b^	82	3	<0.001^*^	1.00
metastasis	18	15		15.72 (4.55-54.40)

Statistical analyses: Kaplan-Meier curve model ^*^p<0.05 ^a^Clinicopathological data of some cases could not be obtained from the patients with cervical invasive cancer because of incomplete records of medical chart. ^b^As a reference. ^c^HR, hazard ratio and 95% CI, 95% confidence interval for tissue inhibitor of metalloproteinases-3 genetic polymorphisms rs9619311, rs9862 and rs11547635 and clinicopathological factors, compared to their respective controls. Survival: +, survival, -, mortality.

**Table 5:** Multivariate analysis of genetic polymorphisms of tissue inhibitor of metalloproteinases-3 and clinicopathological variables for 5 years survival in cervical cancer patients

	5 years survival	
**Variables**	*P* value	HR & 95% CI^b^
***TIMP-3* genetic polymorphisms**		
**rs9619311**		
TC /CC vs. TT^a^	0.741	0.72 (0.11-5.00)
CC vs. TT & TC^a^	0.988	u.a.
**rs9862**		
CT/TT vs. CC^a^	0.644	0.74 (0.21-2.63)
TT vs. CC/CT^ a^	0.774	1.28 (0.24-6.98)
**rs11547635**		
CT/TT vs. CC^a^	0.146	3.05 (0.68-13.68)
TT vs. CC/CT^a^	0.406	0.43 (0.06-3.18)
**Clinicopathological characteristics**		
**Pelvic lymph node**		
metastasis vs. no metastasis^a^	0.017^*^	6.84 (1.41-33.11)

Statistical analyses: Cox proportional hazard model ^*^p<0.05 ^a^As a comparison reference ^b^HR, hazard ratio and 95% CI, 95% confidence interval for tissue inhibitor of metalloproteinases-3 genetic polymorphisms rs9619311, rs9862 and rs11547635 as well as clinicopathological characteristics, compared to their respective controls. TIMP-3, tissue inhibitor of metalloproteinases-3; u.a., unavailable.
